# Electroporator with automatic change of electric field direction improves gene electrotransfer *in-vitro*

**DOI:** 10.1186/1475-925X-6-25

**Published:** 2007-07-02

**Authors:** Matej Reberšek, Cécile Faurie, Maša Kandušer, Selma Čorović, Justin Teissié, Marie-Pierre Rols, Damijan Miklavčič

**Affiliations:** 1 University of Ljubljana, Faculty of Electrical Engineering, Tržaška 25, SI-1000 Ljubljana, Slovenia; 2 Institut de Pharmacologie et de Biologie Structurale du CNRS UMR 5089, 205, route de Narbonne, 31077 Toulouse cedex, France

## Abstract

**Background:**

Gene electrotransfer is a non-viral method used to transfer genes into living cells by means of high-voltage electric pulses. An exposure of a cell to an adequate amplitude and duration of electric pulses leads to a temporary increase of cell membrane permeability. This phenomenon, termed electroporation or electropermeabilization, allows various otherwise non-permeant molecules, including DNA, to cross the membrane and enter the cell. The aim of our research was to develop and test a new system and protocol that would improve gene electrotransfer by automatic change of electric field direction between electrical pulses.

**Methods:**

For this aim we used electroporator (EP-GMS 7.1) and developed new electrodes. We used finite-elements method to calculate and evaluate the electric field homogeneity between these new electrodes. Quick practical test was performed on confluent cell culture, to confirm and demonstrate electric field distribution. Then we experimentally evaluated the effectiveness of the new system and protocols on CHO cells. Gene transfection and cell survival were evaluated for different electric field protocols.

**Results:**

The results of *in-vitro *gene electrotransfer experiments show that the fraction of transfected cells increases by changing the electric field direction between electrical pulses. The fluorescence intensity of transfected cells and cell survival does not depend on electric field protocol. Moreover, a new effect a shading effect was observed during our research. Namely, shading effect is observed during gene electrotransfer when cells are in clusters, where only cells facing negative electro-potential in clusters become transfected and other ones which are hidden behind these cells do not become transfected.

**Conclusion:**

On the basis of our results we can conclude that the new system can be used in *in-vitro *gene electrotransfer to improve cell transfection by changing electric field direction between electrical pulses, without affecting cell survival.

## 1. Background

Gene therapy is an experimental method used in clinics proven to be successful in *in-vitro *and *in-vivo *conditions. For gene therapy, DNA or RNA molecules are transferred into living cells to replace, change or silence gene expression. Consequently cells change their biological nature in therapeutical purposes [1,2]. Effective and potentially safe transfer of DNA molecules into living cells has been a goal of scientific research for many years. This research is now divided into two main fields: viral and non-viral gene delivery. Viral vectors are considered to provide the highest effectiveness of DNA transfer, but they are often associated with immune responses [3] and insertional mutagenesis [4-6]. That is why non-viral methods of DNA transfer are being sought for [7-9].

An exposure of a cell to adequate amplitude and duration of electric pulses leads to temporary increase of cell membrane permeability while preserving cell viability. This phenomenon, termed electroporation or electropermeabilization, allows various otherwise non-permeant molecules to cross the membrane and enter the cell. Both *in-vitro *and *in-vivo*, reversible electropermeabilization allows for internalization of a wide range of substances [10,11]. When DNA molecules are transferred into cells by electropermeabilization, this method is called gene electrotransfer. Gene electrotransfer is therefore a non-viral method used to transfer DNA molecules into living cells by means of high-voltage electric pulses [11-16]. Being extensively investigated, gene electrotransfer is becoming more and more effective and therefore gaining importance as a non-viral gene therapy method [7,9].

Electropermeabilization of the cell occurs in the area of cell membrane facing negative and positive electro-potential regarding intercellular potential [17,18]. However, DNA molecules do not spontaneously interact with mammalian cell membrane but are driven to the membrane by electrophoretic forces. Therefore, negative DNA molecules only interact with the cell membrane facing negative electro-potential. Thus, only one side of cell membrane is susceptible for transfer of DNA molecules. Any increase in the susceptible area for transfer of DNA molecules therefore increases the effectiveness of transfection [19,20].

Changing the electric field direction between electrical pulses presumably increases the area of successful electropermeabilization [21] and therefore increases susceptible area for transfer of DNA molecules. This method is especially effective for cells *in-vivo *and also for plated cells *in-vitro*, because their cell shapes and their orientations in the electric field are important for successful electropermeabilization [22-24]. Changing the polarity of electric field during the electric pulse delivery is also important for gene electrotransfer as it allows interaction of DNA molecules on both sides of the cell membrane perpendicular to direction of electric field (changing the electric field polarity corresponds to changing the electric field direction for 180°). Changing the electric field direction between electrical pulses therefore improves the efficiency of gene electrotransfer indirectly by increasing the area of successful electropermeabilized membrane, or directly by interaction of DNA molecules with the cell membrane on both sides.

The protocol that defines changes of electric field direction between electrical pulses is referred to as the electric field protocol. Researchers who had already investigated influence of electric field protocol on the gene electrotransfer did not use any of the existing systems for automatic change of electric field direction between electrical pulses. That is because they did not have any electrodes which would allow delivery of electric field protocols with relatively homogeneous electric field intensity. Because of that they had to change the electric field direction by rotating the electrodes manually, which however is not always possible [20]. Nevertheless similar research, with or without such automatic system, had also been done in electrochemotherapy, but predominantly with the aim of improving electric field distribution including its homogeneity [21,25].

The aim of our research was to develop and test new system and protocol which would improve gene electrotransfer by automatic change of electric field direction between electrical pulses. For this we chose an electroporator, which can control at least four electrodes. In addition, we designed new electrodes made of four cylindrical rods that provides as homogeneous electric field distribution as possible. We calculated the distribution of electric field numerically for given electrode design and electric field protocol. New system and protocols were tested experimentally on Chinese Hamster Ovary cells. *In-vitro *gene transfection and cell survival were evaluated for different electric field protocols by fluorescence microscopy. A shading effect, previously not yet described in scientific literature was observed during our research.

## 2. Methods

**Figure 1:**
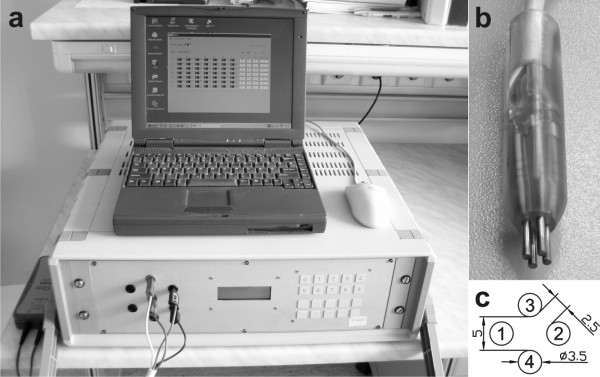
**A new system for gene electrotransfer**. Photograph of the electroporator EP-GMS 7.1 (a), photograph of the electrodes E-S 4.1 (b) and electrodes E-S 4.1 geometry design (c). Electrodes are numbered. Their diameter is 3.5 mm. Electrodes 1 and 2 or 3 and 4 are opposite electrodes and are 5 mm apart. Adjacent electrodes are 2.5 mm apart, which is a half distance between opposite electrodes.

A new system for gene electrotransfer was developed which consists of an electroporator (EP-GMS 7.1, Fig. 1a) and specially designed new electrodes (E-S 4.1, Fig. 1b). Both were developed at the University of Ljubljana, Faculty of Electrical Engineering. The EP-GMS 7.1 electroporator was already used and described in previously reported studies [26,27]. The main advantage of this electroporator is the ability to automatically change the electric field direction between electrical pulses at various frequencies, without rotation or movement of electrodes.

### 2.1 Electroporator (EP-GMS 7.1) and electrodes (E-S 4.1)

The user defines electrical parameters of applied electric pulse through the interface of the electroporator (EP-GMS 7.1) on a personal computer (PC). Parameters are then transferred to the executive part of the electroporator. After this transfer the electroporator is ready to generate defined electric pulses in predefined directions.

Electroporator (EP-GMS 7.1) generates from 1 to 32 square electric pulses from 80 to 400 V, duration from 10 to 1000 μs and repetition frequency from 0.1 to 5000 Hz. Particularity of this electroporator is an embedded electrode commutator which controls up to seven electrodes. This commutator applies one of three possible states to each of the electrodes: positive, negative or high impedance state. Electrode state change is accomplished within 12 ms thus the electric field direction between the electrodes can be changed.

The electrodes (E-S 4.1) were designed as four cylindrical rods that allow delivery of electric field in different directions and at the same time providing relatively homogeneous electric field distribution. Delivery of electric field in all directions can be achieved by two sinusoidal signals phase shifted for 90°, which are delivered on two pairs of opposite electrodes (e.g. 1–2 and 3–4, Fig. 1c). However, for that different electroporator should be used, which allows delivery of such sinusoidal signals.

The electrodes are made of stainless steel; their diameter is 3.5 mm, adjacent electrodes are 2.5 mm apart, opposite electrodes are 5 mm apart, their length is 18 mm (Fig. 1c). Electrodes are connected to 4-wire cable and fixed with polyester resin, which assures constant distance between the electrodes and also protects the user against high-voltage.

**Figure 2:**
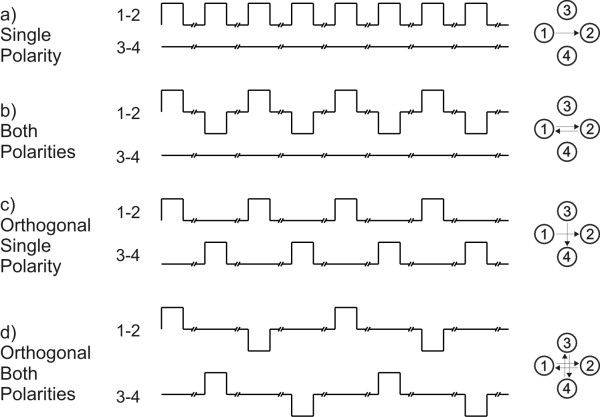
**Electric field protocols**. In single polarity (SP) electric field protocol direct electric pulses are applied between two opposite electrodes. While in both polarities (BP) electric field protocol alternating electric pulses are applied between two opposite electrodes. In orthogonal single polarity (OSP) electric field protocol direct electric pulses are applied between both opposite pairs of electrodes. While in orthogonal both polarities (OBP) electric field protocol alternating electric pulses are applied between both opposite pairs of electrodes. Signals in the middle represent applied voltage to the electrodes. Symbols on the right represent electric field protocols in which arrows represent directions of electric field in the centre between the electrodes.

Four different electric field protocols were used with this new system (electroporator and electrodes) in our experiments: single polarity (SP), both polarities (BP), orthogonal single polarity (OSP) and orthogonal both polarities (OBP; Fig. 2). When SP electric field protocol is used, single polarity electric pulses are applied between two opposite electrodes (Fig. 2a). When BP electric field protocol is used, both polarities electric pulses are applied between two opposite electrodes (Fig. 2b). When OSP electric field protocol is used, single polarity electric pulses are applied alternately between two pairs of opposite electrodes (Fig. 2c). And when OBP electric field protocol is used, both polarities electric pulses are applied alternately between two pairs of opposite electrodes (Fig. 2d).

### 2.2 Electric field intensity

**Figure 3:**
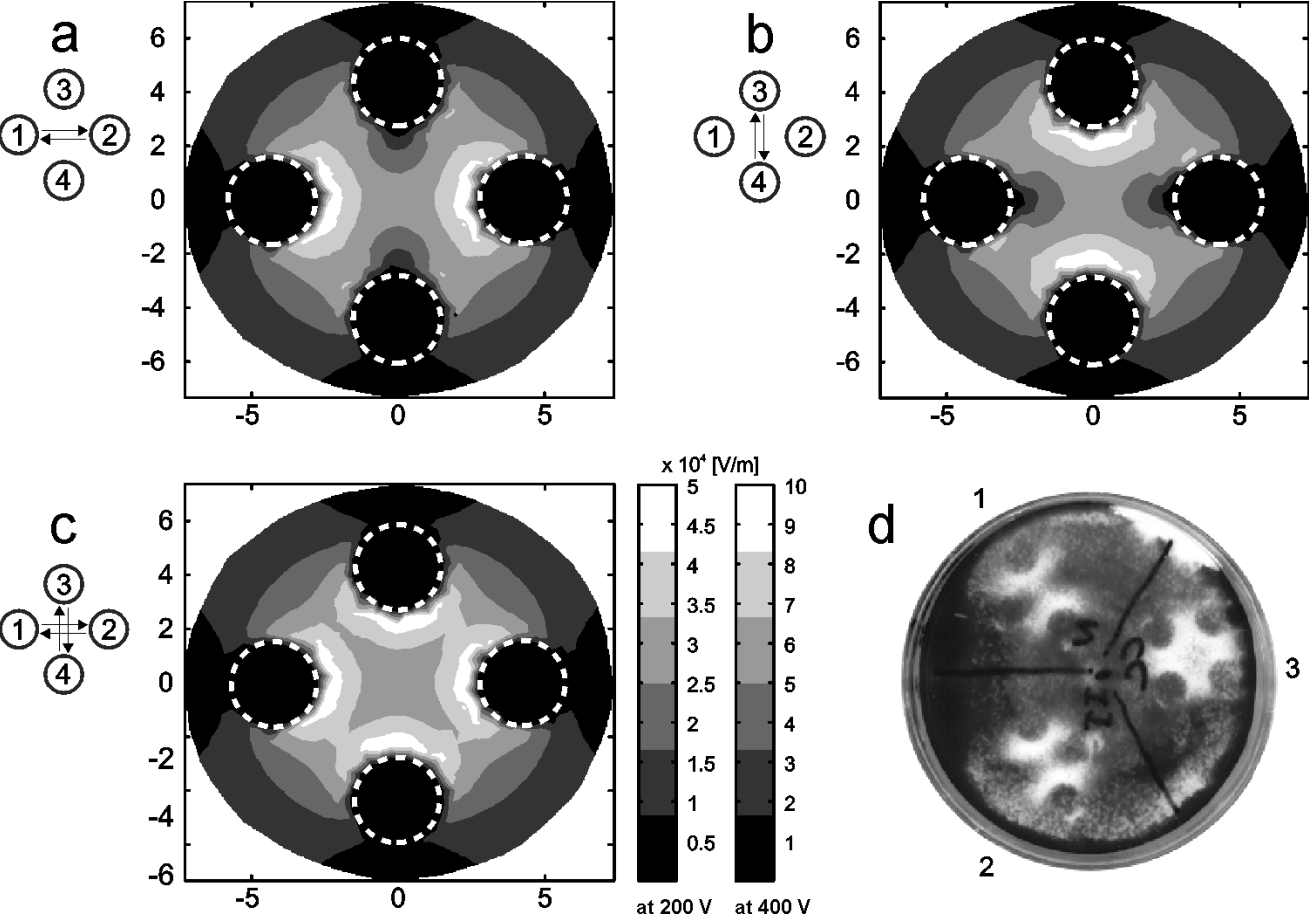
**Calculated electric field intensity between the electrodes**. Calculated electric field intensity between the electrodes during the electric pulse delivery of 200 V (+100 V, -100 V) and 400 V (+200 V, -200 V), when electrical pulses are applied between electrodes 1 and 2 (a) and when they are applied between electrodes 3 and 4 (b). Calculated local maxima of electric field intensity between the electrodes in orthogonal single polarity (OSP) and orthogonal both polarities (OBP) electric field protocol (c). Dashed circles represents position of electrodes. Symbols on the left represent electric field protocols. Electric field intensity scale is given for 200 V and 400 V. Experimental electric field intensity between the electrodes E-S 4.1 during the electric pulse delivery (d), when electrical pulses are applied between electrodes 1 and 2 (d1) and when they are applied between electrodes 3 and 4 (d2). Experimental local maxima of electric field intensity between the electrodes, when electrical pulses are applied between electrodes 1 and 2 and between electrodes 3 and 4 (d3). A train of eight electric pulses with amplitude of 400 V (+200 V, -200 V), duration 1 ms and repetition frequency 1 Hz was applied.

Electric field intensity between the electrodes during the electric pulse delivery was calculated numerically by means of finite-elements method (Fig. 3a–c) [28] and a quick practical test was performed to confirm the correctness of calculations and demonstrate electric field intensity distribution (Fig. 3d).

A three-dimensional finite-elements model of an electroporation medium in culture dish with inserted electrodes (E-S 4.1) was designed using software package EMAS (ANSOFT Corporation, USA). Applied voltage was modelled as Dirichlet's boundary condition on the surface which presents the cross-section of electrode and cell suspension. Electro-potential of disconnected electrodes was defined as zero, because our model is symmetrical and disconnected electrodes are always in the middle between the connected electrodes. Electro-potential of disconnected electrodes was also defined as zero, to satisfy the conditions that electrodes are a lot more conductive then electroporation medium and that the sum of current through the entire surface to disconnected electrodes is always zero. Electroporation medium was mathematically separated from surrounding area by Neuman's boundary condition:

*J*_*N *_= 0,

where J_*N *_is the normal electric current density [A/m^2^]. Electroporation medium was modelled as a constant i.e. independent of electric field applied, passive, homogeneous and isotropic volume conductor in the quasi-stationary electric current field. A condition in such structure is described by Laplace's equation:

Δ*φ *= 0,

where *φ *is electric potential [V]. Results of electric field intensity obtained by such linear model are scalable by applied voltage ratio (Fig. 3).

To calculate the electric field intensity, when electrical pulses are applied between electrodes 1 and 2 (Fig. 3a), boundary conditions on the surface of the electrode 1 were set to +100 V and on the electrode 2 to -100 V. Electrodes 3 and 4 were in this case set to 0 V and thus defined as disconnected. Calculation of electric field intensity, when electrical pulses are applied between electrodes 3 and 4 (Fig. 3b), was done in the similar way as calculation of electric field intensity, when electrical pulses are applied between electrodes 1 and 2. Local maxima of both electric field intensities (Fig. 3c) were calculated to evaluate effectiveness of OSP and OBP electric field protocol [29].

Results of calculations have shown that electric field intensity in the space between the electrodes is very homogeneous compared to electric field intensity between two cylindrical rods, because of the two additional rods, which are highly conductive with respect to electroporation medium, are equalizing the distances between the equipotential lines in the space between the electrodes.

Quick practical test was performed on confluent cell culture in plastic culture dish. To confirm and demonstrate electric field distribution, plastic culture dish was separated into three sections (Fig. 3d). A train of eight electric pulses with amplitude of 400 V (+200 V, -200 V), duration 1 ms and repetition frequency 1 Hz was applied to kill the cells exposed to highest electric field intensity. In the first section electric pulses were applied between electrodes 1 and 2 (Fig. 3d1). In the second section electric pulses were applied between electrodes 3 and 4 (Fig. 3d2). In the third section electric pulses were applied between electrodes 1 and 2 and between electrodes 3 and 4 (Fig. 3d3). After 24 hours, killed cells were washed out and living cells were fixed in plastic culture dish with methanol for 10 minutes and stained with crystal violet.

### 2.3 Cells, cell survival and gene electrotransfer

Chinese Hamster Ovary (CHO; European Collection of Cell Cultures, Great Britain) cells were used. Cells in suspension were cultured in Eagle's Minimum Essential Medium (MEM; Sigma, USA) supplemented with 10 % Foetal Calf Serum (FCS; Sigma, USA). When cell suspension density reached 2 × 10^6 ^cells/ml, it was diluted with culture medium. For experiments, 5 × 10^5 ^cells were plated in a plastic culture dish (growth surface: 9.2 cm^2^, diameter: 40 mm, height: 11 mm; TPP, Switzerland) and grown in the incubator (37°C, 5% CO_2_) for 24 hours. During that time they attached to the surface of the culture dish and started to divide.

For gene electrotransfer experiments, plasmid DNA pEGFP-C1 (Clontech, USA; 4649 base pairs), which expresses green fluorescent protein (GFP, excitation 488 nm, emission 507 nm) under promoter cytomegalovirus, was added in concentration 40 μg/ml to the electroporation medium (10 mM phosphate buffer K_2_HPO_4_/KH_2_PO_4_, 1 mM MgCl, 250 mM sucrose; pH: 7.4, conductivity: 0.14 S/m). Culture medium was removed and 100 μl drop of electroporation medium containing plasmids was placed between electrodes. A train of eight electric pulses with amplitude of 200 V (+100 V, -100 V), duration 1 ms and repetition frequency 1 Hz was applied according to previous results [19,20]. Four different electric field protocols were used as described previously in subsection 2.1: single polarity (SP), both polarities (BP), orthogonal single polarity (OSP) and orthogonal both polarities (OBP) (Fig. 2), to determine gene expression. In the control, cells were not exposed to electric pulses.

After electroporation, cells were left for 15 min at room temperature for cell membrane resealing. Then 2 ml of culture medium was added and culture dishes were then placed into incubator (37°C, 5% CO_2_). 24 hours after electroporation, cells were investigated under inverted fluorescence microscope (Axiovert 200, Zeiss, Germany). Five photos of phase contrast and fluorescence images were taken per sample randomly in the area in the centre between the electrodes with cooled CCD camera (12 bit; VisiCam, Germany). Objective magnification was 20× and approximately 100 cells per image were observed. For fluorescence imaging, excitation wavelength 425 nm (Polycome IV, Visitron Systems, Germany), dichroic mirror (460 DCLP; Chroma, USA) and emission filter (D505/40 m; Chroma, USA) were used.

**Figure 4:**
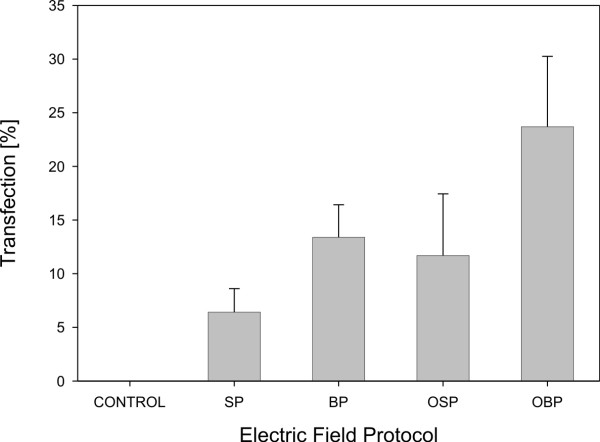
**Fraction of transfected CHO cells**.  Fraction of transfected CHO cells after gene electrotransfer experiment in different electric field protocol. Cells were exposed to a train of eight pulses with amplitude of 200 V, duration 1 ms and repetition frequency 1 Hz. Results were obtained by means of fluorescence microscopy. Each value in the graph represent mean of four independent experiments, ± standard deviation. Electric field protocols result in different fraction of transfected cells (ANOVA: P = 0.002).

**Figure 5:**
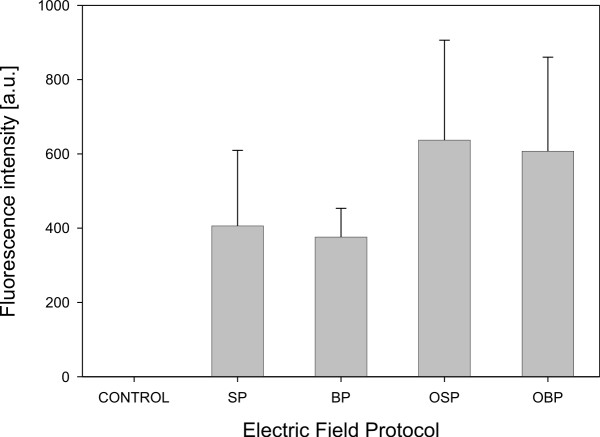
**Fluorescence intensity of transfected CHO cells**. Fluorescence intensity of transfected CHO cells after gene electrotransfer experiment in influence of electric field protocol. Cells were exposed to a train of eight pulses with amplitude of 200 V, duration 1 ms and repetition frequency 1 Hz. Results were obtained by means of fluorescence microscopy. Each value in the graph represent mean of four independent experiments, ± standard deviation. Different electric field protocols did not result in different level of fluorescence intensity (ANOVA: P = 0.246).

MetaMorph (Version 5.0r7, Universal Imaging Corporation, USA) was used for image analysis. The fraction of transfected cells was calculated as the ratio between transfected cells and all viable cells in a given treatment. The fluorescence intensity of transfected cells related to quantity of GFP inside the transfected cells was quantified on acquired images by MetaMorph. The fraction of cell survival was calculated as the ratio between viable cells in treatment and viable cells in control, which were not treated with electric pulses. Independent experiments of gene electrotransfer were repeated four times. Results (the fraction of transfected cells, the fluorescence intensity of transfected cells and the fraction of cell survival) are given in a form of bar graphs (SigmaPlot 9.0, Systat, USA), where every point represents the mean of four independent experiments and the error bars indicate the standard deviation (Fig. 4, 5). Statistical tests One way analysis of variance (One Way ANOVA) were performed on all results (SigmaStat 3.1, Systat, USA). Bonferroni t-test was performed on results if there was indication of a statistically significant difference between different electric field protocols used.

**Figure 6:**
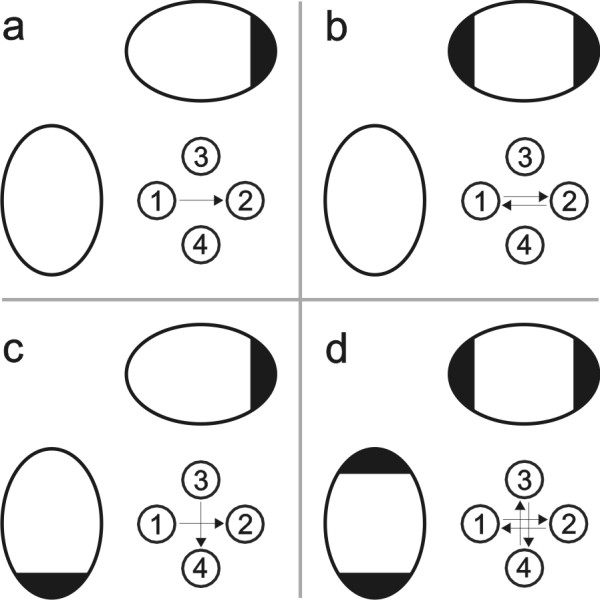
**Schematic drawing of competent areas for DNA interaction with cell membrane**. Schematic drawing of competent cell membrane areas for DNA interaction with cell membrane in influence of electric field protocols: single polarity (a), both polarities (b), orthogonal single polarity (c) and orthogonal both polarities (d). Black areas represent regions of permeabilized membrane where DNA interacts with cell membrane.

**Figure 7:**
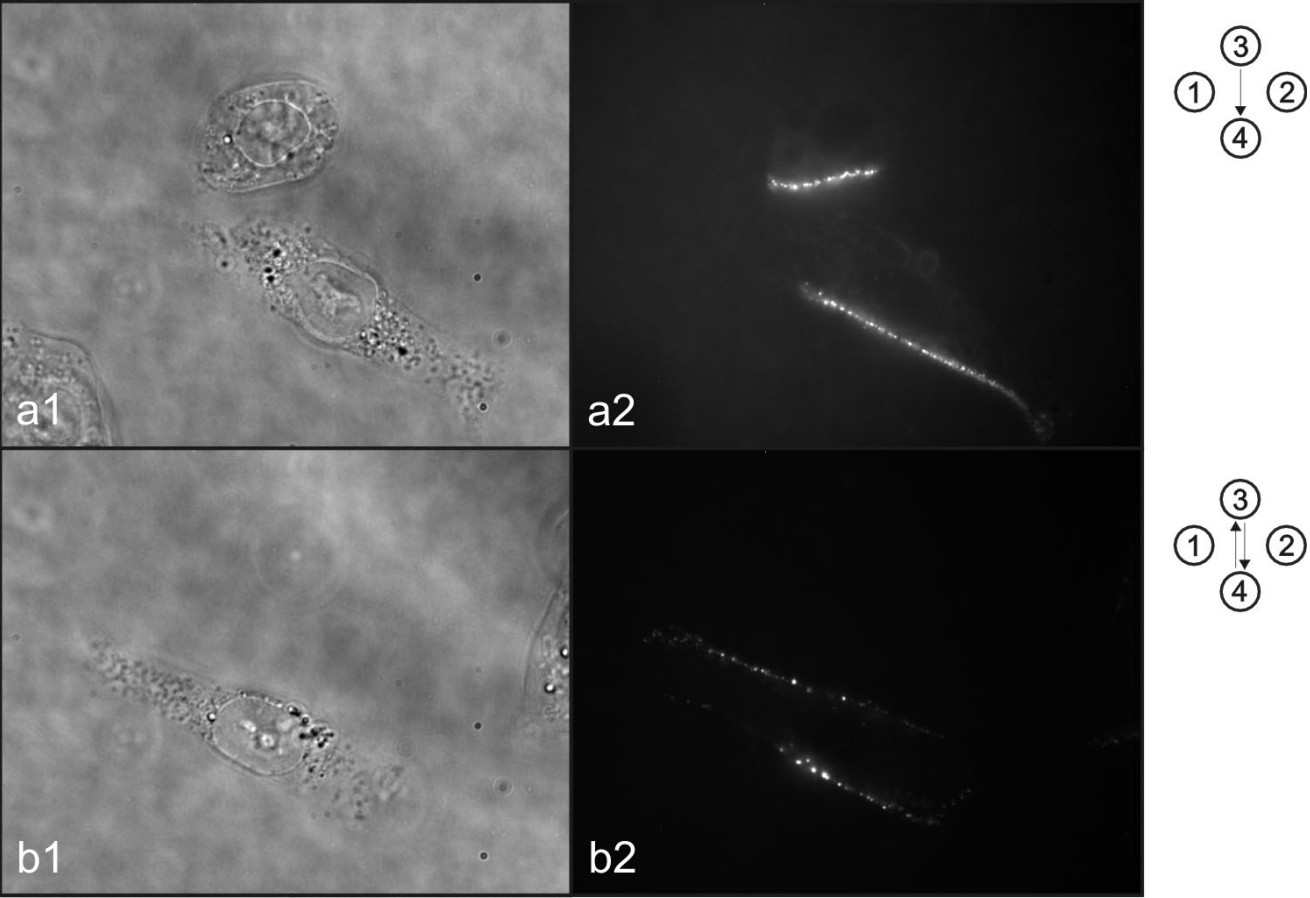
**Visualization of interaction between DNA and cell membrane**. Visualization of interaction between DNA and cell membrane after single polarity electric field protocol (a) and both polarities electric field protocol (b). Photos of phase contrast (1) and fluorescence (2) images were taken under inverted fluorescence microscope. Symbols on the right represent electric field protocol used.

**Figure 8:**
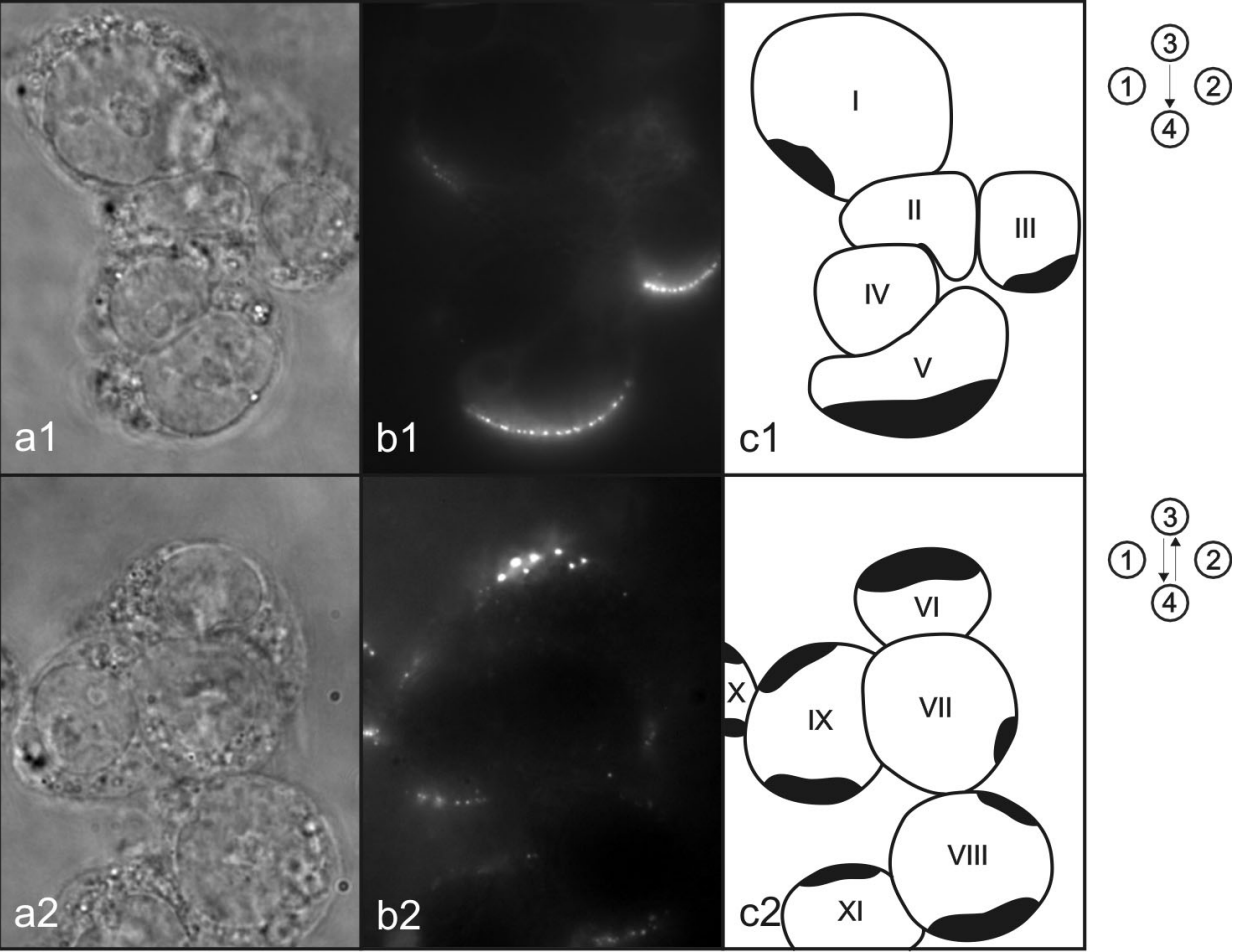
**Shading effect**. Photos of phase contrast (a) and fluorescence (b) images were taken under inverted fluorescence microscope. Symbolic picture (c) was made for better representation of the observed shading effect. Drawn shapes represent cells and black areas represent regions of permeabilized membrane where DNA interacts with cell membrane. Symbols on the right represent electric field protocol used.

To visualize interaction of DNA with cell membrane immediately after application of electric pulses, we stained plasmid DNA pEGFP-C1 with thiazole orange homodimer dye (TOTO-1, excitation 514 nm, emission 533 nm; Molecular Probes, USA). Plasmid DNA pEGFP-C1 was mixed with TOTO-1 by base pair to dye ratio of 5 and placed on ice for 1 hour [30]. Electropermeabilization procedure was the same as for gene electrotransfer, except that only two different electric field protocols were used as described previously in subsection 2.1: single polarity (SP) and both polarities (BP; Fig. 2), to determine areas of DNA interaction with cell membranes (Fig. 6a, b). Up to 5 minutes after electropermeabilization photos of phase contrast and fluorescence images of cells were taken under inverted fluorescence microscope (Fig. 7, 8). For fluorescence imaging excitation wavelength 480 nm (Polycome IV, Visitron Systems, Germany), dichroic mirror (Q505LP; Chroma, USA) and emission filter (HQ535/50m; Chroma, USA) were used.

## 3. Results

Effects of four different electric field protocols: single polarity (SP), both polarities (BP), orthogonal single polarity (OSP) and orthogonal both polarities (OBP) on *in-vitro *gene electrotransfer were evaluated by determining the fraction of transfected cells (Fig. 4) and the fluorescence intensity of transfected cells (Fig. 5). At the same time also the fraction of cell survival was determined.

The results of our *in-vitro *gene electrotransfer experiments show that the fraction of transfected cells increases by changing the electric field direction between electrical pulses. This increase is almost quadrupled at OBP electric field protocol with respect to SP electric field protocol. The largest fraction of transfected cells was observed at OBP electric field protocol and was 24 % (Fig. 4). One way ANOVA indicates that there is a statistically significant difference between different electric field protocols (P = 0.002). Bonferroni t-test indicates that there is a statistically significant difference in comparison of OBP versus SP electric field protocol (P = 0.001) and OBP versus OSP electric field protocol (P = 0.023).

The fluorescence intensity of transfected cells does not however depend on electric field protocol (Fig. 5). One way ANOVA indicated that there is no difference between the electric field protocols (P = 0.246), although transfected cells exposed to orthogonal polarities show higher intensity.

Cell survival after electric pulses applied at 200 V is in the range 96 – 102 % at all four electric field protocols (data not shown). One way ANOVA indicated that there is no difference between the electric field protocols and control (P = 0.963).

Visualization of interaction between DNA and cell membrane showed that DNA molecules interact with the cell membrane facing negative electro-potential (Fig. 7). If SP electric field protocol is used, DNA interacts with cell membrane only from one side of the cell (Fig. 7a) whereas if BP electric field protocol is used, DNA interacts with cell membrane from two sides of the cell (Fig. 7b).

Shading effect is observed when cells are in clusters (Fig. 8). In such clusters we can observe that cells facing negative electro-potential are shading other cells so that DNA molecules can not interact with them (Fig. 8c). Therefore if SP electric field protocol is used the cells in clusters, which are exposed to one side of negative electro-potential during SP electric field protocol, interacts with DNA molecules (III and V, partially: I; Fig. 8c1) and the cells, which are hidden behind this cells in clusters, does not interact with DNA molecules (II and IV, partially: I; Fig. 8c1). And if BP electric field protocol is used the cells in clusters, which are exposed to one of both sides of negative electro-potential during BP electric field protocol, interacts with DNA molecules (VI and VIII, partially VII; Fig. 8c2) and the cells, which are hidden behind this cells in clusters from both sides, does not interact with DNA molecules (partially: VII; Fig. 8c2).

## 4. Discussion and Conclusion

The aim of our research was to develop and test new system and protocol which would improve gene electrotransfer by automatic change of electric field direction between electrical pulses. For this we chose electroporator with embedded electrode commutation circuit, which controls up to seven electrodes and applies one of three possible states to each of the electrodes: positive, negative or high impedance. Although any other electroporator could be used for such experiments. Since previous observations already demonstrated that homogeneity of electric field distribution affects the effectiveness of electropermeabilization [29,31], we developed electrodes that allow as homogeneous electric field distribution as possible.

An ideal homogeneous electric field distribution can only be achieved between two infinite flat electrodes. In practice we achieve a very close approximation to such electric field distribution if we use sufficiently large flat parallel electrodes that are relatively close to each other. But between two parallel electrodes only two directions of electric field are possible. To generate electric field in more that two directions we need to use more electrodes. We could use four plate electrodes, but in this case we get very inhomogeneous electric field distribution between the electrodes, since the current predominantly flows through the metal of the adjacent electrodes and less through the sample (cells suspension or tissue). That is why in the development of new electrodes (E-S 4.1) we focused on conductivity between opposite electrodes and between adjacent electrodes. Our hypothesis in the development of electrodes was that the most homogeneous electric field distribution between four cylindrical electrodes is achieved when conductivity between opposite electrodes is twice the sum of conductivity between adjacent electrodes (Fig. 1c).

To evaluate homogeneity of electric field between the electrodes, we designed a three-dimensional finite-elements model of an electroporation medium in culture dish with inserted electrodes. Calculations of electric field intensity in this model showed that the electric field distribution is relatively homogeneous between the electrodes for all four different electric field directions (Fig. 3). Results of calculations have also shown that orthogonal single polarity (OSP) and orthogonal both polarities (OBP) electric field protocols are efficient only in the space between the electrodes, because only there the electric field direction can be rotated for 90°. In addition, a quick practical test was performed to confirm the correctness of calculations and demonstrate electric field intensity distribution (Fig. 3d). In this test, cell survival was depended on electric field intensity. At highest electric field intensity all cells where killed and at low electric field intensity all cells survived. Good agreement was obtained between calculated and experimental data.

In the next step we experimentally evaluated effectiveness of new system and electric field protocols to improve *in-vitro *gene electrotransfer on Chinese Hamster Ovary cells. Results show that changing the electric field direction between electrical pulses increases the fraction of transfected cells (Fig. 4), with no statistically significant influence on fluorescence intensity of transfected cells (Fig. 5) and cell survival. Therefore, the results obtained in our research support previous observations that changing the electric field direction between electrical pulses improves gene electrotransfer with no significant effect on cell survival [20,32].

Plated cells are of various shapes and often elongated. Because of this, their orientation in electric field is important [23]. If they are elongated in the direction of electric field, they have higher probability to be permeabilized and that DNA interacts with this part of the membrane (Fig. 6a, b). If we change the direction of electric field during electric pulse delivery, cumulatively more cells are elongated in the direction of electric field and therefore DNA interacts with the membranes of more cells (Fig. 6c, d). Consequently there are more transfected cells (Fig. 4).

We observed another effect during our research i.e. a shading effect, which is also important for efficient gene electrotransfer of plated cells. Shading effect is observed during gene electrotransfer when cells are in clusters, where only cells facing negative electro-potential in clusters become transfected and other ones which are hidden behind these cells do not become transfected (Fig. 8c1). And if we change electric field direction between electrical pulses, cumulatively more cells face negative electro-potential (in case of inhomogeneous electric field distribution the direction of electric field is not always the same as direction towards electrodes, therefore the term facing electro-potential is used instead of the term facing electrodes) in cluster and more cells in cluster become transfected (Fig. 8c2). Therefore changing electric field direction between electrical pulses improves fraction of transfected cells in clusters, which is another reason why it is advisable to use orthogonal both polarities (OBP) electric field protocol instead of single polarity (SP) electric field protocol.

For each electric field protocol we used the same cumulative number of pulses (Fig. 2). Thus, if we used more directions of electric field during electric pulse delivery, fewer pulses were delivered in each direction. Therefore from each direction a lower "degree" of membrane permeabilization is obtained and less DNA interacts with the cell membrane. But overall, our results indicate that fluorescence intensity of transfected cells is not affected when using our protocols.

Cell survival was also not significantly affected by electric pulse application, which is important for gene transfection as damaged cells difficultly express genes [33]. This means that our protocol is also appropriate for cells which are valuable, such as human primary cells, which are taken directly from a donor or patient [34].

On the basis of our results we can conclude that although homogeneity of electric field distribution between the newly designed electrodes presented in this paper is not as good as between two parallel plate electrodes, the results of gene electrotransfer are improved. By automatic change of electric field direction, electric pulses can be delivered at precise frequencies, which enables new experiments for better understanding of DNA interaction with cell membrane. In addition, the new system can be used wherever manual rotating is not possible, like in case of multiple electrodes, when they are used with different electric field direction between electrical pulses. Such an embedded electrode commutator is being built in the Cliniporator device [35]. In conclusion, the main advantage of the new system and electric field protocol is that it can be used in *in-vitro *gene electrotransfer to improve fraction of transfected cells without affecting fluorescence intensity of transfected cells and cell survival by using automatic orthogonal both polarities electric field protocol.
